# Effects and implications of the COVID-19 pandemic on medicine use by employees of a Brazilian public university: a cross-sectional study

**DOI:** 10.1590/1516-3180.2021.0367.R1.23072021

**Published:** 2022-02-07

**Authors:** Waléria de Paula, Wanessa Cecília de Oliveira, Adriana Lúcia Meireles, Renata Cristina Rezende Macedo do Nascimento, Glenda Nicioli da Silva

**Affiliations:** I MSc. Doctoral Student, Postgraduate Program on Pharmaceutical Sciences (CiPharma), School of Pharmacy, Universidade Federal de Ouro Preto (UFOP), Ouro Preto (MG), Brazil.; II Undergraduate Biology Student, Institute of Exact and Biological Sciences, Universidade Federal de Ouro Preto (UFOP), Ouro Preto (MG), Brazil.; III PhD. Professor, Department of Clinical and Social Nutrition, Postgraduate Program on Health and Nutrition, Universidade Federal de Ouro Preto (UFOP), Ouro Preto (MG), Brazil.; IV PhD. Professor, Department of Pharmacy, Postgraduate Program on Pharmaceutical Sciences (CiPharma), School of Pharmacy, Universidade Federal de Ouro Preto (UFOP), Ouro Preto (MG), Brazil.; V PhD. Professor, Department of Clinical Analysis, Postgraduate Program on Pharmaceutical Sciences (CiPharma), School of Pharmacy, Universidade Federal de Ouro Preto (UFOP), Ouro Preto (MG), Brazil.

**Keywords:** Drug utilization, COVID-19, Universities, Faculty, Pharmacoepidemiology, Medicine use, University professors, Technicians

## Abstract

**BACKGROUND:**

During the COVID-19 pandemic, universities have had to adopt remote education, a strategy that caused sudden changes of routine for everyone involved in academia.

**OBJECTIVE:**

To assess the profile of medicine use by the employees of a Brazilian public university during the COVID-19 pandemic.

**DESIGN AND SETTING:**

Cross-sectional study at a Brazilian public university.

**METHODS:**

Employees were invited to answer an online self-administered questionnaire, containing questions on sociodemographic features, medicine use, mental health and lifestyle habits during the COVID-19 pandemic. The outcome variable was the use of medicines stratified according to occupation. Descriptive, bivariate and multivariate (Poisson regression) statistical analyses were performed.

**RESULTS:**

A total of 372 employees participated in the study and use of medicine was reported by 53.2%. Among professors, suicide attempts (prevalence ratio [PR], 1.81; 95% confidence interval [CI], 1.20-2.74), physical activity (PR, 1.53; 95% CI, 1.11-2.11) and poor self-rated health (PR, 1.29; 95% CI, 1.01-1.66); and among technicians, decreased workload during the COVID-19 pandemic (PR, 1.41; 95% CI, 1.00-1.99), excess body weight (PR, 1.39; 95% CI, 1.02-1.88) and poor self-rated health (PR, 1.48; 95% CI, 1.14-1.92) were positively associated with use of medicines. In addition, among technicians, engaging in physical activity (PR, 0.60; 95% CI, 0.46-0.78) was a protective factor against medicine use.

**CONCLUSION:**

The profile of medicine use among these employees was similar to that of the Brazilian population. However, some associated factors may have been influenced by the COVID-19 pandemic, thus highlighting the need to examine this topic in a longitudinal study.

## INTRODUCTION

The COVID-19 pandemic, caused by exponential transmission of the severe acute respiratory syndrome coronavirus 2, has had serious consequences worldwide.^1,2^ In Brazil, since the first case was reported, the number of infections and deaths has been increasing, reaching 18,557,141 confirmed cases on June 30, 2021, at 7 pm. The average numbers of cases and deaths have tended to fluctuate considerably among Brazil’s states and regions. However, the southeastern region currently (2021) has the highest moving average of COVID-19 cases.^3^

In order to reduce the transmission rates, some coping measures have been adopted, such as quarantine or social distancing,^4,5^ and Brazilian universities have adopted distance learning. This strategy has caused sudden changes to the routines of all people involved in academia.^6^

In this context, professors and technicians who carry out face-to-face activities with students have had to adapt to a new reality and develop new teaching skills. These changes associated with social distancing may have affected these employees’ mental health because they normally interact with different people as part of their workload. Additionally, the feeling of anxiety related to an unstable future may have led to mental health issues among those who faced such situations.^7,8^ This situation had the potential to increase their use of psychotropic medicines. In addition, data from the Federal Pharmacy Council (Conselho Federal de Farmácia, CFF) indicated that the use of psychotropic medicines during the pandemic (January to July 2020), especially medicines in the anticonvulsant and antidepressant classes, increased by 12.8% and 13.84%, respectively.^9^

To the best of our knowledge, no study has explored the use of medicines among employees of higher education institutions during the COVID-19 pandemic and whether the profile of medicine use by this specific population is similar to that of the general adult population.

## OBJECTIVE

The aim of the present study was to assess the profile of medicines consumed by the employees of a Brazilian public university during the COVID-19 pandemic and the factors associated with this use of medicines.

## METHODS

### Study design and participants

This cross-sectional study was integrated with a project on anxiety and depression among university students (PADu) that was conducted by researchers at the Universidade Federal de Ouro Preto (UFOP), titled “Effect of the COVID-19 pandemic on mental and nutritional health and on the home food environment of the academic community: longitudinal evaluation - PADu-COVID”. Currently, UFOP has 11,993 students enrolled in 55 undergraduate courses, 726 technicians (administrative and laboratory technicians) and 934 professors. The study population consisted of all technicians and professors at UFOP.^10^

For this article, only employees (university professors and technicians) were evaluated, and the participants were recruited through a virtual invitation. This constituted convenience sampling, in which all employees were invited by email to participate and the sample consisted of those who answered the questionnaire.

### Ethical issues

All potential participants were asked to read and agree to the informed consent statement online before they could participate in the study. A copy of this statement was made available for download. This step was necessary before they could start answering the questionnaire. The PADu-COVID project received ethical approval under number CAAE 31077320.7.1001.5150 on June 28, 2020.

### Data collection

The employees’ e-mail addresses were obtained through the UFOP Information Technology Center. These e-mail addresses were used with the university’s consent. Information about this study was also spread via the university’s website, social networks, unions and academic directories that supported the project.

All UFOP employees were invited to participate in the study between July 20 and August 27, 2020, through virtual invitations sent out by email and through social network channels, using snowball sampling. The questionnaire was made available on an online platform (Google Forms) and the invitations were sent out once a week, on alternate days, over a period of one month.

### Outcome and explanatory variables

The questionnaire consisted of questions on sociodemographic characteristics, medicine use, mental health, lifestyles and health conditions during the COVID-19 pandemic. The outcome variable of the study was the use of medicines by the employees. The data on this variable was obtained through the question: “In the last 30 days, have you taken any medicine?”. The answer options were “no” or “yes”. If the response was yes, the participant was asked to report the name of the medicine. Subsequently, the medicines reported were grouped into classes in accordance with the second level of Anatomical Therapeutic Chemical (ATC).^11^

The explanatory variables were the following: age (< 45 years or ≥ 45 years); sex (female or male); sexual orientation (heterosexual, homosexual, bisexual or asexual); housing situation (living alone, with family/relatives, in student housing or with friends); skin color (white; or others: East Asian, brown or black); marital status (single/divorced/widowed, married or common-law marriage); religious belief (no or yes); family income (up to six minimum monthly wages in Brazil or greater than or equal to six minimum monthly wages in Brazil); decreased income due to the pandemic (no, yes or yes, more than 50%); symptoms of anxiety (no or yes), depression (no or yes), stress (no or yes) or suicidal ideation (no or yes), or suicide attempts (no or yes) or social distancing (no or yes); workload (still the same, increased or decreased); alcohol consumption (no or yes) and alcohol consumption since the pandemic started (still the same, increased or decreased); smoking (never smoked, quit smoking or currently smoking); illicit drug use (no or yes); engaging with physical activity (no or yes); body mass index (no excess body weight or excess body weight); and self-rated health (good or poor).

The monetary values for the monthly minimum wage were presented in Brazilian reais, converted into United States dollars (USD) using the purchasing power parity (PPP) method. Thus, the value of 1 USD was equivalent to 2.25 Brazilian reais, according to the conversion rate for the year 2019.^12^

### Statistical analyses

The data from the survey questionnaire were automatically stored in a database in the Excel software, 2019 version (Microsoft, Washington, United States) and were coded. Statistical analyses were performed using the STATA 13.0 software (StataCorp LLC, Texas, United States), namely:

1. Descriptive analysis according to frequencies.
2. Bivariate analysis according to Pearson’s chi-square and Fisher’s exact tests, to determine associations between the outcome of interest, i.e. medicine use stratified according to occupation, and the sociodemographic, mental health, lifestyle and health condition variables during the COVID-19 pandemic. Only the explanatory variables that showed an association with a level of significance of ≤ 0.20 in the bivariate analysis were included in the subsequent multivariate analysis.
3. Multivariate analysis using Poisson regression was carried out to determine the possible factors associated with the use of medicine. For this analysis, a significance level of 0.05 was adopted. In the multiple regression model, the data entry method of backward selection was used, i.e. all the variables selected in the bivariate analysis were inserted at the same time in the model and were removed one-by-one, starting from the least significant. The final model was adjusted for age and sex.

## RESULTS

A total of 372 employees participated in the study: 213 university professors and 159 technicians. Most of the participants were younger than 45 years (66.1%), female (56.4%), heterosexual (90.9%), living with their family (76.4%), of self-declared white skin color (59.4%) and married (60.0%); and had a religious belief (71.0%) and a family income greater than or equal to six minimum monthly wages in Brazil (81.4%). In addition, 60.7% of the participants did not experience any decrease in family income during the COVID-19 pandemic (**Table 1**).

**Table 1.** Descriptive analysis on sociodemographic variables, medicine use, mental health conditions and lifestyle habits during the COVID-19 pandemic, among employees of the Universidade Federal de Ouro Preto, Ouro Preto, 2020 (n = 372)

Variables	n	%
Sociodemographic characteristics		
**Age**		
< 45 years	246	66.1
≥ 45 years	126	33.9
**Occupation**		
University professor	212	57.0
Technicians	169	43.0
**“Gratified” functions**		
No	322	86.6
Yes	50	13.4
**Sex**		
Female	210	56.4
Male	162	43.6
**Sexual orientation**		
Heterosexual	338	90.9
Homosexual, bisexual, and asexual	34	9.1
**Housing situation**		
Living alone	57	15.3
With family/relatives	284	76.4
In student housing or with friends	31	8.3
**Skin color**		
White	221	59.4
Others (East Asian, brown or black)	151	40.6
**Marital status**		
Single/divorced/widowed	149	40.0
Married or common-law marriage	223	60.0
**Religious belief**		
No	108	29.0
Yes	264	71.0
**Family income**		
Up to six minimum monthly wages in Brazil	69	18.6
Greater than or equal to six minimum monthly wages in Brazil	303	81.4
**Income decrease due to pandemic**		
No	226	60.7
Yes	142	38.2
Yes, more than 50%	4	1.1
Use of medicines		
**Were medicines used?**		
No	174	46.8
Yes, 1-4	188	50.5
Yes, 5 or more	10	2.7
Cardiovascular medicines		
No	328	88.2
Yes	44	11.8
**Nervous system medicines**		
No	309	83.1
Yes	63	16.9
Mental health		
**Symptoms of anxiety**		
No	307	82.5
Yes	65	17.5
**Symptoms of depression**		
No	287	77.2
Yes	85	22.8
**Symptoms of stress**		
No	288	77.4
Yes	84	22.6
**Suicidal ideation**		
No	314	84.4
Yes	58	15.6
**Suicide attempt**		
No	365	98.1
Yes	7	1.9
Lifestyle and health conditions during the COVID-19 pandemic		
**Social distancing**		
No	22	5.9
Yes	350	94.1
**After the start of the pandemic, your workload was:**		
Still the same	140	37.6
Increased	79	21.3
Decreased	153	41.1
**Alcohol consumption**		
No	123	33.1
Yes	249	66.9
**After the start of the pandemic, your alcohol consumption was:**		
Still the same	160	64.3
Increased	34	13.6
Decreased	55	22.1
**Smoking**		
No, I never smoked	305	82.0
I quit smoking	39	10.5
I currently smoke	28	7.5
**Illicit drug use**		
No	356	95.7
Yes	16	4.3
**Physical activity**		
No	103	27.7
Yes	269	72.3
**Body mass index**		
No excess body weight	202	54.3
Excess body weight	170	45.7
**Self-rated health**		
Good SRH	308	82.8
Poor SRH	64	17.2

One Brazilian minimum monthly wage was equivalent to 464.44 United States dollars, when converted according to purchasing power parity (PPP $) in 2019.^12^ Common-law marriage: long-lasting, public affective relationship between two individuals who want to build a family, as acknowledged through the Brazilian Federal Constitution.^13,14^ SRH: self-rated health. “Gratified” functions are those involving direction, leadership, advisory or secretariat functions, among others, which give rise to additional payments.

Regarding the use of medicines, 53.2% of the employees took some medicine, among whom 2.7% used five or more medicines simultaneously, known as polypharmacy. Furthermore, the professors reported using 183 medicines, of which 37 (20.2%) were prescribed after the onset of the COVID-19 pandemic; the technicians reported using 149 medicines, of which 25 (16.8%) were prescribed after the onset of the pandemic. Psychotropic medicines were the most frequently used medicines, especially after the onset of the pandemic, and antiepileptics (N03), antidepressants (N06) and analgesics (N02) were the most frequently cited pharmacological classes.

**Figure 1.**
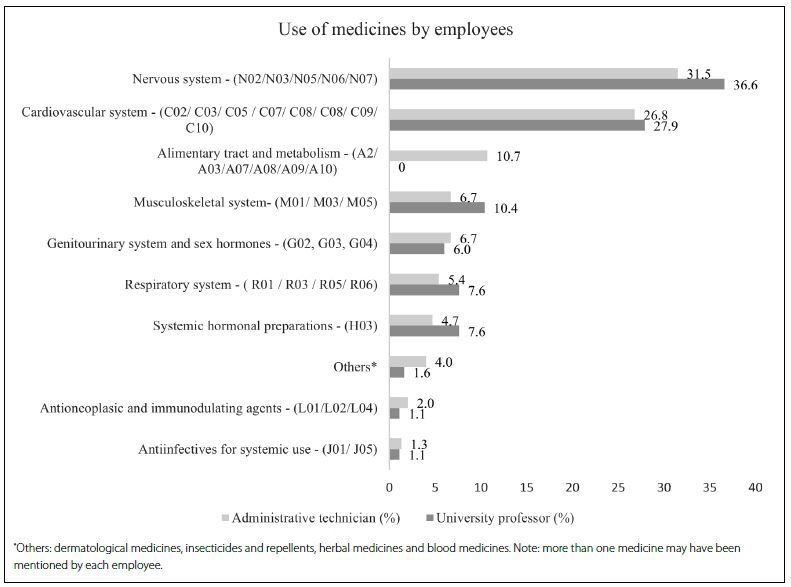
Prevalence of medicine use among technicians and professors, according to the first and second levels of Anatomical Therapeutic Chemical (ATC).^11^

Antidepressants were the most widely used medicines. Among the professors and technicians, the prevalences of antidepressant use were 13.7% and 16.8%, respectively. Analgesics were also widely cited by the participants, at a prevalence rate of 9.3% among the professors and 10.1% among the technicians. Among cardiovascular system medicines, the most frequently cited ones were angiotensin II antagonists, at a prevalence of 11.5% among the professors and 10.1% among the technicians. The percentages of medicines used, in accordance with the first and second levels of Anatomical Therapeutic Chemical (ATC), by professors and technicians, are presented in **Figure 1**. Medicines for the nervous and cardiovascular systems were the ones most frequently reported by the professors and technicians.

The frequencies of medicine use, stratified according to occupation, in relation to sociodemographic, mental health and lifestyle variables during the COVID-19 pandemic, are shown in **Table 2**.

**Table 2.** Bivariate analysis on the prevalence of medicine use, stratified according to occupation, in relation to sociodemographic variables, medicine use, mental health conditions and lifestyle habits during the COVID-19 pandemic among employees of the Universidade Federal de Ouro Preto, Ouro Preto, 2020 (n = 372)

Variables	Use of medicines			
	University professor (%)	P	Technicians (%)	P
Sociodemographic characteristics				
**Age**				
< 45 years	46.6	**< 0.001**	42.6	**0.009**
≥ 45 years	71.9		65.9	
**“Gratified” functions**				
No	56.3	0.911	46.4	**0.083**
Yes	55.2		66.7	
**Sex**				
Female	61.3	0.280	62.6	**< 0.001**
Male	50.0		30.9	
**Sexual orientation**				
Heterosexual	53.6	0.055*	49.3	0.846
Homosexual, bisexual and asexual	84.2		46.7	
**Housing situation**				
Living alone	48.3	0.611*	32.1	**0.123**
With family/relatives	57.1		51.7	
In student housing or with friends	64.3		58.8	
**Skin color**				
White	54.8	0.503	53.3	0.308
Others (East Asian, brown or black)	59.7		45.2	
**Marital status**				
Single/divorced/widowed	50.7	0.253	47.5	0.693
Married or common-law marriage	59.0		50.6	
**Religious belief**				
No	52.7	0.435	38.2	**0.155**
Yes	58.3		52.0	
**Family income**				
Up to six minimum monthly wages in Brazil	60.0	0.619*	45.3	0.438
Greater than or equal to six minimum monthly wages in Brazil	56.2		51.6	
**Income decrease due to pandemic**				
No	56.6	0.361*	43.3	0.091*
Yes	57.3		55.2	
Yes, more than 50%	0.0		100.0	
Mental health				
**Symptoms of anxiety**				
No	54.	**0.103**	44.5	**0.020**
Yes	67.6		67.7	
**Symptoms of depression**				
No	54.7	0.340	47.0	0.389
Yes	62.8		54.8	
**Symptoms of stress**				
No	56.5	0.905	45.0	**0.073**
Yes	55.6		61.5	
**Suicidal ideation**				
No	53.4	**0.049**	50.7	0.303
Yes	71.4		39.1	
**Suicide attempt’**				
No	55.3	0.055*	49.0	0.742*
Yes	100.0		50.0	
Lifestyle and health conditions during the COVID-19 pandemic				
**Social distancing**				
No	60.0	0.540*	58.3	0.357*
Yes	56.2		48.3	
**After the start of the pandemic, your workload was:**				
Still the same	54.0	0.885	39.4	**0.091**
Increased	58.1		52.1	
Decreased	57.4		60.0	
**Alcohol consumption**				
No	59.1	0.587	59.6	**0.046**
Yes	55.1		43.1	
**After the start of the pandemic, your alcohol consumption was:**				
Still the same	54.8	0.307*	46.3	0.661
Increased	70.6		35.3	
Decreased	48.6		38.9	
**Smoking**				
No, I never smoked	56.3	0.684*	48.8	0.925
I quit smoking	61.5		53.8	
I currently smoke	46.1		46.7	
**Illicit drug use**				
No	56.2	0.540	49.7	0.359*
Yes	60.0		33.3	
**Physical activity**				
No	41.8	**0.012**	72.9	**< 0.001**
Yes	61.4		38.7	
**Body mass index**				
No excess body weight	51.7	**0.128**	36.9	**0.001**
Excess body weight	62.1		62.7	
**Self-rated health**				
Good SRH	53.4	**0.060**	40.9	**< 0.001**
Poor SRH	70.3		88.9	

One Brazilian minimum monthly wage was equivalent to 464.44 United States dollars, when converted according to purchasing power parity (PPP $) in 2019.^12^ Common-law marriage: long-lasting, public affective relationship between two individuals who want to build a family, as acknowledged through the Brazilian Federal Constitution.^13,14^SRH: self-rated health. “Gratified” functions are those involving direction, leadership, advisory or secretariat functions, among others, which give rise to additional payments. *Value obtained through Fisher’s exact test.

In the bivariate analysis (**Table 2**), statistically significant differences were observed between university professors who used medicines and the variables of age, suicidal ideation and physical activity. Among the technicians, this association occurred in relation to the variables of age, sex, symptoms of anxiety, alcohol consumption, physical activity, body mass index and self-rated health.

The results from the multivariate analysis for medicine use stratified according to occupation were adjusted for age and sex in order to minimize possible confounding factors (**Table 3**). Among the professors, suicide attempts (prevalence ratio [PR], 1.81; 95% CI, 1.20-2.74), physical activity (PR, 1.53; 95% CI, 1.11-2.11) and poor self-rated health (PR, 1.29; 95% CI, 1.01-1.66) were positively associated with the use of medicines.

**Table 3.** Multivariate analysis using Poisson regression adjusted for sex and age, according to the occupation of employees of the Universidade Federal de Ouro Preto, Ouro Preto, 2020 (n = 372)

Variables	Use of medicines			
	University professor		Technicians	
	PR (95% CI)	P	PR (95% CI)	P
**Suicide attempt**				
No	1	0.005	–	–
Yes	1.81 (1.20-2.74)		–	
**After the start of the pandemic, your workload was:**				
Still the same	–	–	1	
Increased	–		1.19 (0.84 - 1.68)	0.316
Decreased	–		1.41 (1.00 - 1.99)	0.047
**Physical activity**				
No	1	0.009	1	< 0.001
Yes	1.53 (1.11-2.11)		0.60 (0.46 - 0.78)	
**Body mass index**				
No excess body weight	–	–	1	0.035
Excess body weight	–		1.39 (1.02-1.88)	
**Self-rated health**				
Good SRH	1	0.040	1	0.003
Poor SRH	1.29 (1.01-1.66)		1.48 (1.14-1.92)	

SRH = self-rated health; PR = prevalence ratio; 95% CI = 95%

Among the technicians, decreased workload during the COVID-19 pandemic (PR, 1.41; 95% CI, 1.00-1.99), excess body weight (PR, 1.39; 95% CI, 1.02-1.88) and poor self-rated health (PR, 1.48; 95% CI, 1.14–1.92) were associated with increased medicine use. On the other hand, physical activity (PR, 0.60; 95% CI, 0.46-0.78) was negatively associated with medicine use, thus indicating that it was a protective factor against medicine use (**Table 3**).

## DISCUSSION

To the best of our knowledge, this was the first study to assess the use of medicines by the employees of a Brazilian public university during the COVID-19 pandemic. Important results were found for this population.

The results showed that there was high prevalence of medicine use among these employees (53.23%). Corroborating our findings, the National Survey on Access, Use and Promotion of Rational Use of Medicines of 2015 (Pesquisa Nacional de Acesso, Utilização e Promoção do Uso Racional de Medicamentos, PNAUM), which investigated the use of medicines in a representative sample of the Brazilian population, found an overall prevalence of medicine use of 50.70%.^15^ In addition, other studies on adult populations found similar overall prevalences of medicine use.^16,17^ In our study, medicines for the nervous and cardiovascular systems were the ones most cited, as also observed in other studies involving adult populations.^18,19^

de Mesquita Mororó et al. analyzed population-based data from the National Health Survey and found that access to medicines is unequal between the regions of Brazil and that higher education levels are related to increased access.^20^ This aspect of education, as a determinant of access to medicines, has also been found in other studies.^21,22^ However, despite the fact that access to medicines is linked to their profile of use, the prevalence of medicine use among the population in the present study was similar to that reported in studies involving the adult population in general.

For these university professors and technicians, the prevalence of use of new medicines after the pandemic started was around 31%. Use of psychotropic medicines was frequently reported, with emphasis on antiepileptics, analgesics and antidepressants. High use of medicines for mental health issues is a common response to moments of crisis, such as the COVID-19 pandemic. In a systematic review conducted by Xiong et al. (2020), the negative impacts on the mental health of individuals during this period were highlighted, especially the indications for pharmacological treatment.^23^ Thus, the COVID-19 pandemic may have contributed to the beginning of the use of this class of medicines in the UFOP academic community.

Chronic non-communicable diseases, such as hypertension, diabetes mellitus and other comorbidities, are known to be risk factors for the occurrence of severe symptoms of COVID-19.^24–26^ In the present study, medicines for the cardiovascular system formed the second most reported class. Use of some cardiovascular medicines, such as angiotensin-converting enzyme (ACE) inhibitors and angiotensin receptor blockers may be associated with severe COVID-19 infection.^25,27^ Mehra et al. (2020) indicated that cardiovascular diseases themselves and not the use of medicines are related to increased mortality among patients hospitalized with COVID-19. Thus, several independent authorities have recommended that the use of these medicines should not be interrupted among patients with COVID-19, considering the absence of robust clinical evidence.^28^

The use of medicines during the pandemic by the professors and technicians was associated with several socioeconomic, mental health, lifestyle and physical health condition characteristics during the COVID-19 pandemic. Among the professors, suicide attempts, physical activity and poor self-rated health were associated with the use of medicines.

Many professors experienced mental illnesses during the COVID-19 pandemic, as they entered an environment of great pressure and had to adapt to new technological and digital resources quickly, while reconciling remote work and personal life in the same environment.^29^ Stress in the work environment is associated with occurrence of mental disorders and can be a risk factor for use of antidepressants.^30^

Physical activity was associated with increased use of medicines among the professors. The association between physical activity and increased medicine use may have been influenced by the presence of chronic cardiovascular diseases among the professors and the recommendations to remain active to ensure good quality of life.^31,32^

Poor self-rated health was associated with increased use of medicines among the professors. In a systematic review carried out during the COVID-19 pandemic, Vindegaard and Benros (2020) observed that negative self-assessment of health can lead to an increased risk of psychiatric symptoms or worsening of psychological wellbeing, which consequently leads to use of medicines.^33^ In addition, the presence of comorbidities was also associated with negative self-rated health.^34^

For the technicians, the decrease in the workload during the pandemic, excess body weight and poor self-rated health were associated with increased use of medicines, while physical activity was a protective factor against use of medicines. The decrease in the workload, which occurred in the first months of the COVID-19 pandemic, coincided with the period of data collection. This variable was associated with increased use of medicines among the technicians. During this period, the employees had to adapt to a new scenario; they also had to face a loss of daily routines and impaired personal and social contacts. In addition, the pandemic period was accompanied by the emergence of heightened emotions, especially the fear of contracting or transmitting COVID-19. When academic activities were being organized for distance learning, this may have provided some leisure initially, but it eventually led to high levels of stress and anxiety while waiting for new ways of working.^35,36^ Consequently, it may have become necessary to start pharmacological treatment in order to minimize psychological damage.

Another factor associated with the use of medicines among technicians was excess body weight. Overweight and obesity are related to the presence of chronic diseases such as cardiovascular diseases.^37,38^ As a large percentage of the technicians reported using cardiovascular medicines, this may explain the association between being overweight and use of medicines. Similar to the professors’ profile of use, poor self-rated health was associated with increased medicine use among technicians. This relationship can be explained by the fact that, in most cases, the medicine use was directly related to disease conditions, thus resulting in poor self-perception of health.^39,40^

Physical activity was associated with reduced use of medicines among the technicians. It is known that physical activity prevents the onset of numerous diseases and, consequently, it may have been a protective factor. The relationship between engagement with physical activity and good health has been widely described in the literature.^41,42^ Good physical and mental health may reduce the need for psychotropic medications.

Although these two classes of employees showed medicine use profiles similar to that of the general population, there were differences in the characteristics associated with the use of medicines, such as the relationship between suicide attempts and use of medicines among the professors and between decreased workload and increased use of medicines among the technicians. Such associations may have been impacted by the COVID-19 pandemic. In order to better understand the profile of medicine use and the differences between these employees, it is necessary to continue the present study in a longitudinal manner.

This study had a cross-sectional design, so the influence of memory bias when reporting medicine use in the last 30 days cannot be ruled out. Data relating to additional occupations such as participation in working groups or postgraduate programs were not collected. In addition, there are many challenges in collecting online data, such as limitations on access to and reliability of the internet network, high non-response rate, selection bias toward those who have access to the internet and familiarity with the platform used for the research. The response rate was 22.6%, which was similar to what is expected for studies involving online surveys.^43,44^ Despite these challenges, online studies have proven to be an important alternative for conducting research during the COVID-19 pandemic.^45^

Lastly, this was an innovative study that highlighted the importance of studies involving employees of educational institutions, especially regarding medicine use and health status, risks of intoxication and addiction, masked symptoms and delayed diagnoses.^46,47^

## CONCLUSION

The results from this study showed that there was high prevalence of medicine use among the professors and technicians at an important public university in Brazil. Although the population studied had a high level of education, the prevalence of medicine use was similar to that of the general population in Brazil. However, it is worth emphasizing that different factors were associated with medicine use among the university professors and technicians, compared with the adult population in general, such as suicide attempts and decreased workload.

Although studies on medicine use are still very scarce, they are very important for understanding the profile of the active user population, demonstrating the role of medicines as necessary inputs for healthcare actions, rationalizing costs and improving the quality and case resolution capacity of the local healthcare system. Educational institutions should seek to understand the circumstances of their employees and should carry out pharmaceutical counseling with regard to rational use of medicines during and after the COVID-19 pandemic. In addition, studies in which it is sought to understand the effects of the pandemic on medicine use need to be continued, considering that irrational use of medicines can lead to health risks.
